# Experimental investigation of Taylor-Couette-Poiseuille flow at low Taylor and Reynolds numbers

**DOI:** 10.1371/journal.pone.0212728

**Published:** 2019-04-03

**Authors:** Magdalena Kristiawan, Mouhammad El Hassan, Alioune El Faye, Václav Sobolík

**Affiliations:** 1 INRA, UR 1268 BIA, BP 71627, Nantes, France; 2 Faculty of Engineering, Prince Mohammad Bin Fahd University, Al Khobar, KSA; 3 LaSIE, UMR 7356, University of La Rochelle, La Rochelle, France; Auburn University, UNITED STATES

## Abstract

Flow patterns of a Taylor-Couette-Poiseuille flow were studied at low axial Reynolds and rotational Taylor numbers (*Re* ≤ 10.5, *Ta* ≤ 319). The radius ratio of the inner and outer cylinders was 0.804 and the ratio of the length of the annulus to the gap width was 44.5. Complete map of the studied flow regimes was elaborated. The axial and azimuthal components of the wall shear rate *γ* were measured at the outer fixed cylinder using a three-segment electrodiffusion probe. The components of the wall shear rate of helices have never been measured in previous investigations. Spatio-temporal description of multiple flow patterns was obtained using flow visualizations and simultaneous measurements of wall shear rate components. The flow structures include Taylor vortices, helices winding in the same direction as the base flow or in the opposite direction, helices that were stagnant or moving in the axial direction, smooth or with superposed azimuthal waves, among others. The influence of different flow structures on the wall shear stress components is discussed with and without axial base flow. It was found that the wall shear stress is a function of *Ta* but no significant dependence on *Re* was observed for the studied flow regimes and that the mean wall shear stress increases with the number of azimuthal waves. It was also noted that the ED probes provide a more detailed information about flow patterns than torque measurements and visualizations described in the literature.

## Introduction

Taylor-Couette-Poiseuille flow (TCPF) due to an axial pressure gradient between a rotating inner cylinder and a fixed outer cylinder exhibits a multitude of regimes. The study of the flow instabilities that lead to various regimes is interesting from the theoretical point of view. However, there are also practical applications of TCPF in separation devices, journal bearing and rotating machinery [1]. Fénot et al.[2] published a review on heat transfer in TCPF. Kataoka et al. [3] used the electrodiffusion technique to study the effect of an axial flow on the local mass transfer coefficients and concluded that the axial flow decreased the mass transfer coefficient. Resende et al. [4] studied mass transfer in a TCPF reactor. Using the Reynolds stress modeling approach Poncet et al. [5] simulated a velocity field and heat transfer in a TCPF with η = 0.961.

The base laminar spiral TCPF becomes unstable as the rotational rate of the inner cylinder is increased. The flow regimes depend on Taylor and Reynolds numbers, which are defined as

$$Ta=\frac{R_{1}\Omega d}{\nu }$$ (1)

$$Re=\frac{u_{m}d}{\nu }$$ (2)

where *u*_*m*_ is the mean velocity of the axial flow, *d* the space between the cylinders, Ω the angular velocity of the inner cylinder with a radius *R*_1_ and ν the kinematic viscosity.

Chandrasekhar [6] and DiPrima [7] studied axisymmetric disturbances for a radius ratio close to 1. They found that an axial flow stabilized the Couette flow, i.e. the transition to Taylor vortices occurred at higher Taylor numbers. The assumption of non-axisymmetric disturbances leads to the appearance of pairs of helical vortices for Reynolds numbers above a critical value. The number of helices *n* can be a positive or negative integer [8]. A positive *n* means that the inclination of the helical vortices is opposite to that of the base spiral flow. Pairs of toroidal counter-rotated vortices exist for *n* = 0 and there are single, double, triple and quadruple helices composed of pairs of counter-rotated spirals for *n* = 1, 2, 3, and 4, respectively. In the theoretical study for radius ratios η from 0.1 to 0.95, Chung and Astill [9] found positive *n*, which was in agreement with the experimental results of Snyder [10], who measured critical *Ta* as a function of *Re* in a small gap apparatus (η = 0.95).

Using the experimental procedure of adjusting axial flow in a completely fulfilled annular gap and then slowly increasing the rotational rate of the inner cylinder (start experiment), Lueptow et *al*. [11] observed helical vortices moving in the direction of the axial flow which had “a negative helix angle with respect to the direction of the flow “(*n* = 1). These vortices existed in a narrow domain of Taylor numbers for Reynolds numbers greater than 8 in the vicinity of the Couette-Poiseuille flow. For higher *Ta*, the helical vortices became wavy and still moved in the direction of the axial flow. Other flow regimes observed by these authors [11] at small *Ta* were wavy vortices and random wavy vortices. They also observed stationary helical vortices with a negative *n* (-2) in a very small region around *Ta* = 150 and *Re* = 6. This flow regime was not mentioned in the next paper concerning the PIV measurements of radial and axial velocities in a meridional plane [12], nor in the numerical simulation results of Hwang and Yang [13].

Bühler [14] studied TCPF in a device with a radius ratio of 0.8. He found helical vortices with values of *n* = -1, -2 and -3. The helical vortices characterized by *n* = -1 were either stationary or moving up or down as a function of *Re*. The helical vortices with *n =* -2 and -3 were always stationary.

Gu and Fahidy [15] did not observe helical vortices in the geometry characterized by η = 0.607–0.893. They described only inclined and wavy inclined vortices with periodical variations of the inclination angle.

Recktenwald et *al*. [16] calculated the critical values of the amplitude equation for the first instability of the Couette-Poiseuille flow over a wide range of radius ratios for small values of *Re* (*Re* < 20). Altmeyer et al. [17] presented marginal stability curves for *Ta* in the interval (0, 300) and *Re* = 6. They found *n* = ±1 and ±2 helices and also an “island” of a *n* = 2 helix in a domain of *n* = -2.

Raguin and Georgiadis [18] reconstructed a three-dimensional velocity field of a stationary helix by means of analytical approximations applied to the experimental data obtained via magnetic resonance imaging. In an experimental set-up with the inner cylinder rotating, axial through-flow and η = 0.5 they observed one pair of spirals (*n* = -1) at *Ta* = 147.2 and *Re* = 11.14. They did not show data about the rotation and axial velocity of the helix.

Using numerical simulation, Martinand et al. [19, 20] found helices (n = 1 ÷ 4) in TCP flow with superimposed radial flow.

Research efforts have also concentrated on the flow in the gap between two cylinders rotating at a different velocity [21–23]. Even if there is no base axial flow, given the similarity of the flow structures which can occur in this configuration, we shall present a brief outline of these papers. Andereck et al. [24] observed laminar spirals, interpenetrating spirals and wavy spirals. Altmeyer et al. [25] elucidated transitions between Taylor vortices and helices in a system with rigid non-rotating lids fixed at the bottom and top of a TC system. Heise et al. [26] experimentally proved that rotating end walls affect the bifurcation scenario. They found asymmetric spiral modes and different mixed states.

Degouchi and Altmeyer [27] numerically simulated different modes and their competitions. They found several interactions of two spirals or Taylor vortex modes. Abcha et al. [28] measured the radial and axial velocity component in Taylor and helical vortices using PIV.

Knowing the wall shear rate or stress is very important for practical applications and also for the verification of numerical simulations of TCPF. The mean value of the azimuthal wall shear stress τ can be calculated from the measured torque [29, 30] or estimated from PIV and LDA measurements [31, 32]. However, the data on local values of wall shear rate is scarce [3, 33–35].

In the present paper we studied the flow regimes which occur in TCPF at low Taylor and Reynolds numbers. The geometry and velocity of flow structures was depicted in films taken with a camera and from the data obtained by an array of electrodiffusion probes flush mounted into the wall of the outer steady cylinder. A three-segment electrodiffusion probe provided us with the wall shear rate and its axial and azimuthal components.

The originality of the present paper consists in the measurement of the shear rate components on the wall of the outer cylinder since the axial component of the wall shear stress of helices was never measured in previous studies. On the other hand, the Taylor-Couette flows consisting in the addition of a mean—annular Poiseuille—flow along the direction of the axis of the cylinders was far less studied than the one without axial flow, and is of interest for practical applications of this rotating flow. Finally, the present investigation focuses on cases slightly beyond the critical conditions above which the laminar flow is destabilized and toroidal or helical vortices develop, then exhibiting some waviness along the azimuthal direction as the control parameter (the rotation of the inner cylinder) is further increased. The description of the vortex dynamics and its influence on the wall shear stress components is a significant addition to the existing literature.

## Experimental setup and measurement methods

### Experimental apparatus

**Fig 1:**
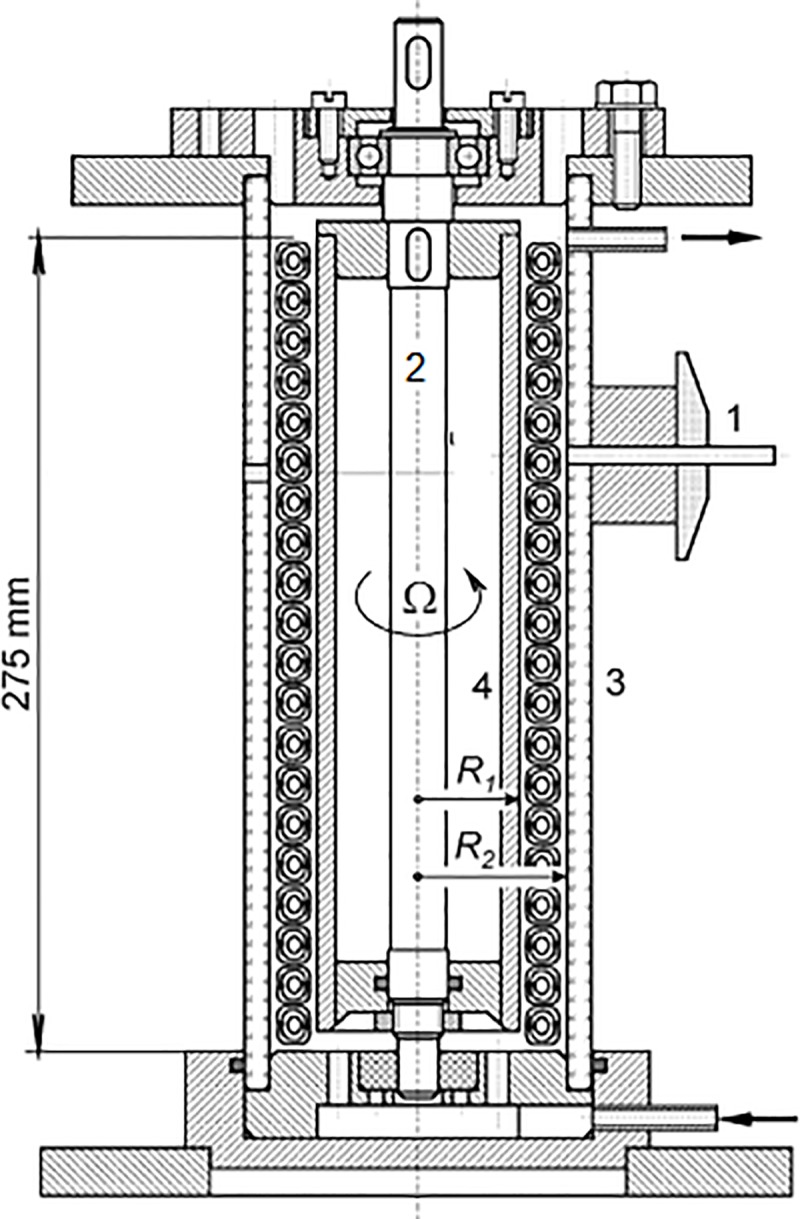
Experimental set-up with Taylor vortices. (1) three-segment electrodiffusion probe, (2) shaft, (3) outer cylinder, (4) inner cylinder. The arrows represent flow input and output.

The experimental apparatus is shown in Fig 1. It consisted of an outer cylinder (3) made of a Plexiglas tube with an inner radius *R*_*2*_ = 30.90 ± 0.05 mm and an interchangeable inner Plexiglas cylinder (4 in Fig 1). The inner cylinder has a length of 275 mm and a radius of *R*_*1*_ = 24.85 ± 0.02 mm. A cylinder with an outer radius of 29.55 ± 0.02 mm was used for the calibration of the electrodiffusion probes in a laminar Couette flow. This allowed us to achieve higher wall shear rates even in a laminar Couette flow. The corresponding radius ratios, η *= R*_*1*_*/R*_*2*_, were 0.804 and 0.956 and the gap width *d* equal to 6.05 and 1.35 mm. The aspect ratio (the height of the cylindrical gap divided by its width *d* = 6.05 mm) was 45.5. The inner cylinder was mounted on a stainless steel shaft (5) which had an upper ball bearing and bottom polyamide sliding bearing. The shaft was driven by a stepping motor with a step of 0.9° and a gear box with a slow-down ratio of 1:9. There was a plastic clutch between the shaft and gear box which also served as electrical insulation. The rotations were controlled directly by the measuring software on a computer.

The axial flow was adjusted with a gear pump. We used either a GJ-N23.FF1S.B.B1 head (Micropump, 0.64 mL/rev.) or a DGS.11 head (Tuthill, 0.11 mL/rev.) driven by Reglo Z (Ismatec). The test liquid was pumped from a small tank with a volume of one liter into an inlet tube in the bottom of the apparatus. The liquid was distributed below the inner cylinder through four holes with a diameter of 4 mm. A spillway was created with a tube mounted 8 mm below the cover and connected to the tank by a hose. The tank was immersed in water at a controlled temperature. Taylor and Reynolds numbers were determined with a precision of 0.5 and 2%, respectively.

### Electrodiffusion method

The measurement of wall shear rate γ by means of the limiting diffusion current is a well known technique [36]. A two electrode cell consisting of a small working electrode and a large auxiliary electrode, a solution containing depolarizer and an excess of supporting electrolyte is sufficient for measuring the limiting diffusion current. The applied voltage must have a value at which only the active species react on the working electrode; their concentration on this electrode is negligibly small. The analogous problem of heat transfer was first solved by Leveque [37]. According to this solution, the current density *i* is given by the relation

$$i(x)=0.5384\;k\;F\;c_{0\;}D^{2/3}\gamma^{1/3}x^{-1/3}$$ (3)

where *x* is measured along a streamline from the front edge of the electrode, *k* is the number of electrons taking part in the reaction, *F* is the Faraday constant and *c*_0_ the concentration in the bulk.

The total current is calculated by integration of the current density over the whole electrode surface. It holds for a circular electrode with a radius *R*:

$$I=2.157\;k\;F\;c_{0}\;D^{2/3}R^{5/3}\gamma^{1/3}.$$ (4)

The wall shear rate was very small in our experiments; hence the condition of a high Peclet number was not satisfied. The exponent on γ was lower than 1/3 and it was necessary to replace Eq 4 by an empirical relation

$$I=K_{j}\gamma^{nj}$$ (5)

where the coefficients *K*_j_ and *n*_j_ were obtained by the probe calibration in the laminar Couette flow. In order to increase the accuracy of the measurements, the interval of γ was divided into two subintervals (*j* = 2). The three-segment probe was calibrated before each series of measurements and the calibration was verified at the end of the series. The error in γ measurements was lower than 6%.

**Fig 2:**
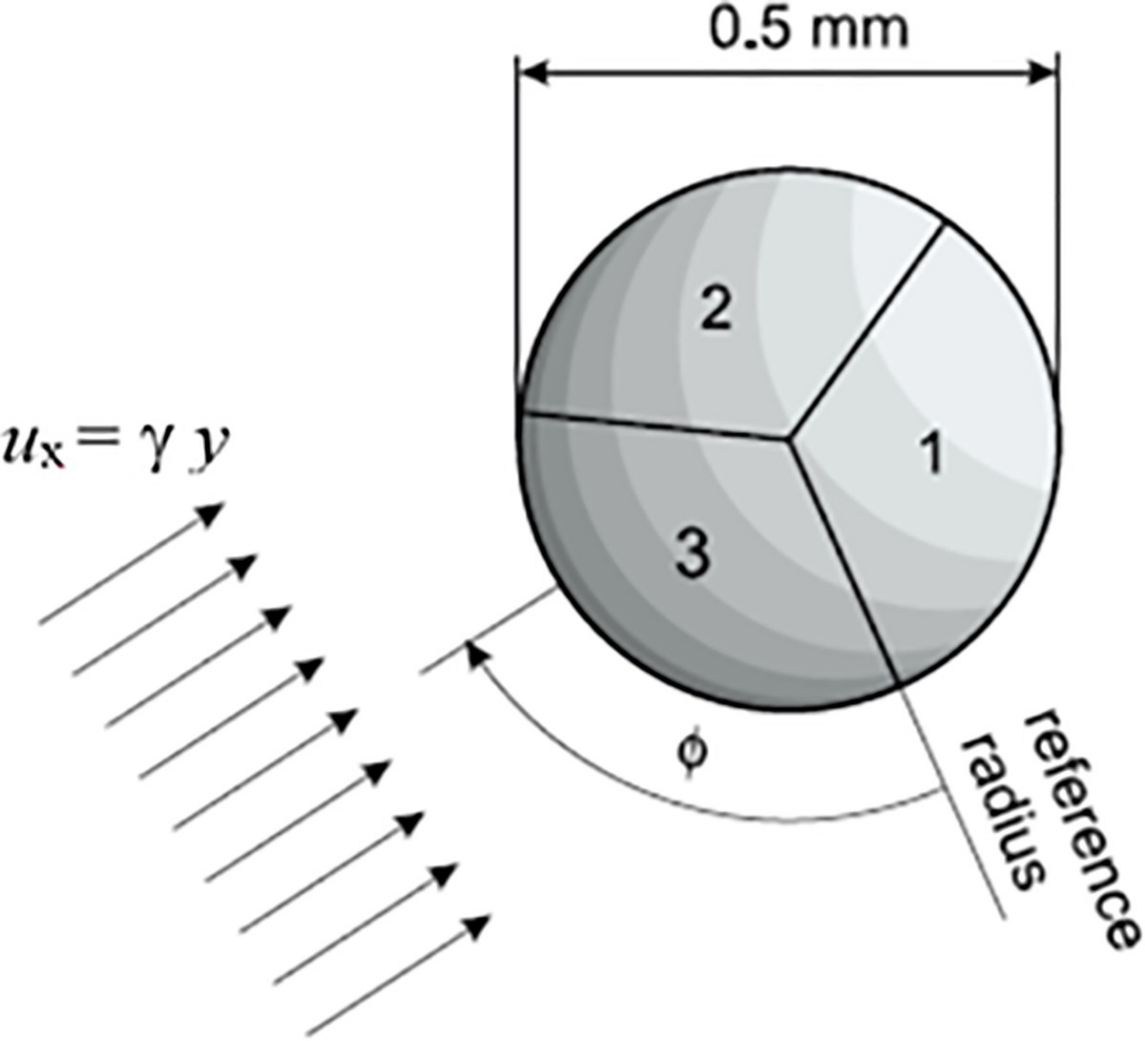
Front view of ideal three-segment probe with current density.

According to Eq 3 the current density decreases with *x* ^*-1/3*^. This fact makes it possible to measure flow direction by using segmented probes composed of several electrically insulated parts. Three-segment probes are capable of flow angle resolution in the range of 360° [38]. The front view of an ideal three-segment probe is shown in Fig 2.

The intensity of current density is denoted by shading in Fig 2: the darker the shading the higher the current density. If the wall shear rate is uniform and the Peclet number high enough, the ratio of current through the segments depends only on the flow direction ϕ. The dependence of the limiting diffusion currents through segments, *I*_*i*_, normalized by the sum of the currents, *I*_*tot*_, on the flow direction *ϕ* is called directional characteristics. These are used to calculate the flow angle from the measured electric currents. The measurement of directional characteristics was carried out during the probe calibration in the laminar Couette flow. The accuracy of flow angle measurements was better than 4°.

**Fig 3:**
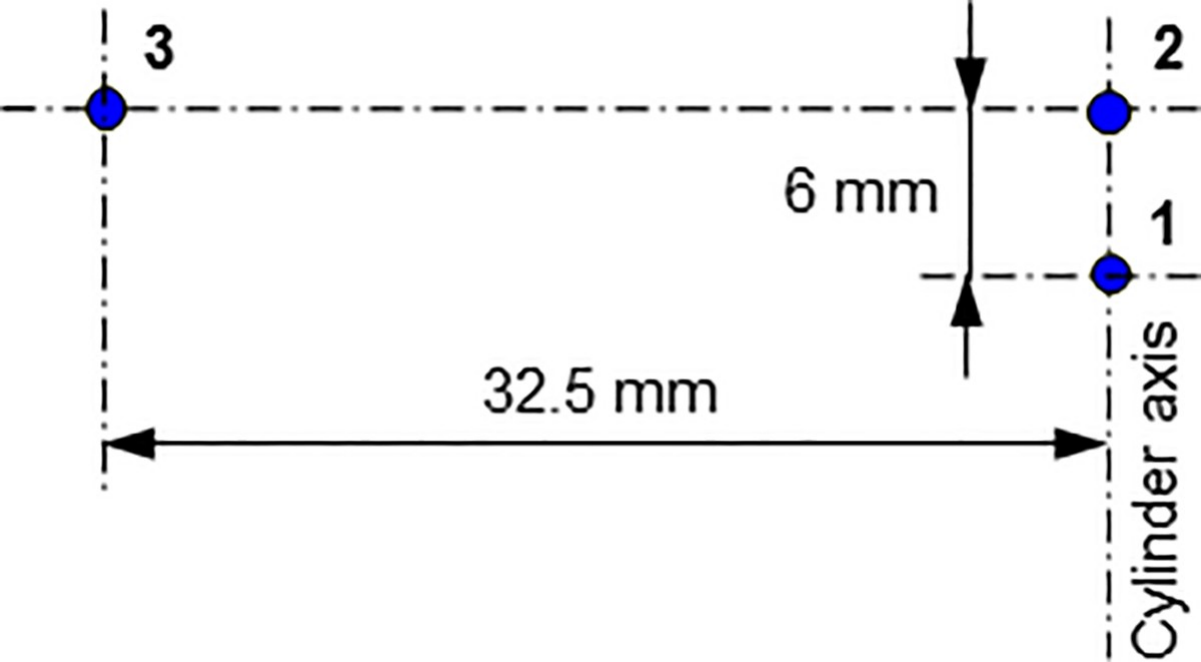
Arrangement of three simple probes with a diameter of 0.1 mm.

A three-segment electrodiffusion (ED) probe (diameter 0.5 mm) and three simple probes (diameter 0.1 mm) were embedded in the inner wall of the outer cylinder. The simple probes were used for the study of flow structures (see Fig 3).

The probes were connected to a six-channel electrodiffusion analyzer, which applied a polarization voltage to the electrodes and converted the currents flowing through the electrodes into voltages. The analyzer was controlled by a PC via an A/D and D/A card. A voltage of -0.8 V was applied between the working electrodes (cathodes) and a large auxiliary electrode (anode).

The test liquid was a 25 mol m^-3^ equimolar potassium hexacyanoferrates (III) and (IV) aqueous solution (*kF* = 96485 C mol^-1^) with 1.5% b.w. K_2_SO_4_ as supporting electrolyte. The flow was visualized by addition of a few drops of AQ-1000 rheoscopic liquid (Kalliroscope Corp., U.S.A.). The rheoscopic liquid contains small laminae reflecting light in a way that depends on their orientation, which follows the flow direction. Films were taken using a Panasonic Lumix FZ8 camera at a rate of 30 frames/s.

## Results and discussion

### Preliminary definitions

The term vortex is used for closed rings (*n* = 0) and the term helix for spiral flows. The base spiral flow consists of a rotation about the vertical axis, which is from left to right (counter clockwise when seen from the top) and the axial flow is oriented upwards from the bottom. Helices which wind in the direction of the base flow were denoted by *n* < 0 in conformity with the literature. Helices with the opposite pitch were defined by *n* > 0. The flow structures rotate in the azimuthal plane and along the cylinder axis and translate in the axial direction.

If a helix rotates around its axis, an observer fixed in the laboratory frame saw axial movement even without axial base flow. It is like a nut on a screw. As the screw shaft rotates, the nut moves linearly along the shaft [39]. We shall call it rotation effect. In our configuration the axial phase speed of helices winding in the same direction as the base spiral flow was downward whereas that of helices with the opposite pitch was upward. The axial phase speed of helices is composed of the translation due to the axial base flow and due to the rotation effect.

It should be noted that the axis of the experimental set-up is horizontal on all films, which does not correspond to the actual setup. Actually, the camera was rotated by 90° about the horizontal axis to maximize the spatial resolution. Therefore, the actual bottom is shown on the left, the spillway on the right and the axial flow is from left to right.

**Fig 4:**
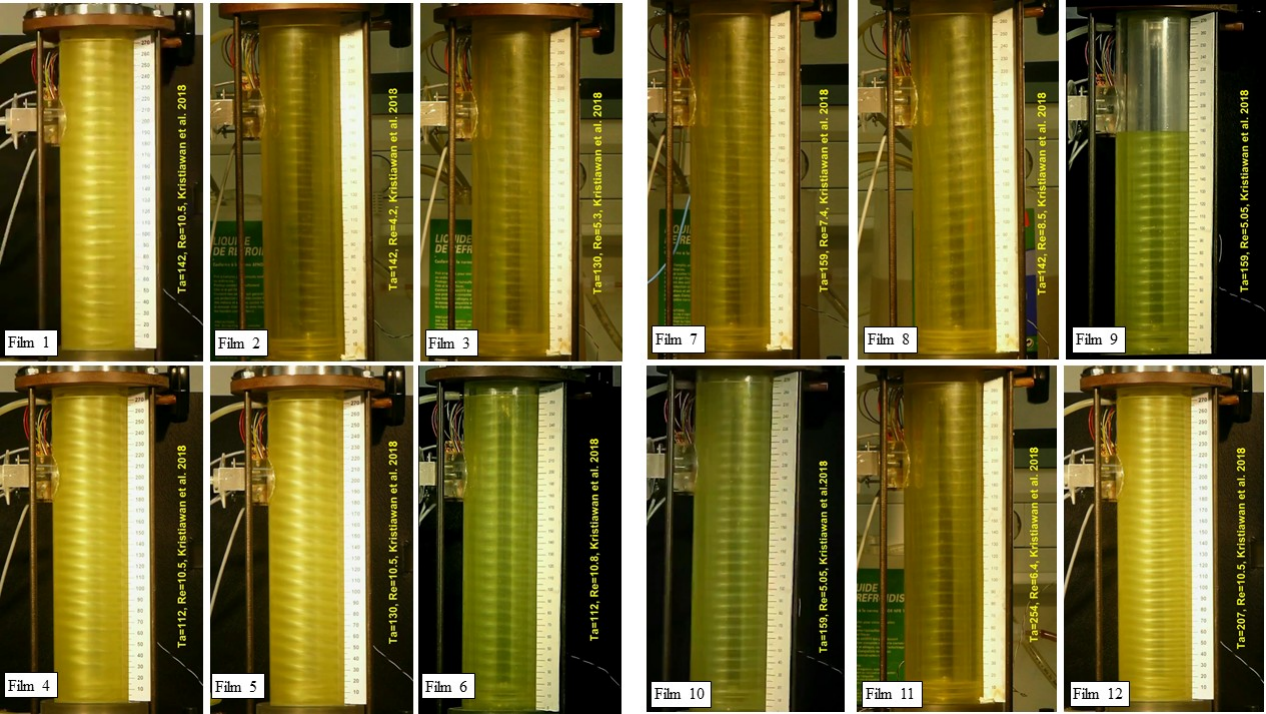
Snapshots of films acquired in the present investigation. For regime abbreviations see Fig 5. Film 1. TV, Ta = 142, Re = 10.5; Film 2. 1V, Ta = 142, Re = 4.2; Film 3. 1+1, Ta = 130, Re = 5.3; Film 4. 0+1, Ta = 112, Re = 10.5; Film 5. 0+2, Ta = 130, Re = 10.5;Film 6. 0+1, Ta = 112, Re = 10.8. Film 7. T+1, Ta = 159, Re = 7.4; Film 8. 0–2, Ta = 142, Re = 8.5; Film 9. 0–1, Ta = 159, Re = 5.05. Film 10. M-1, Ta = 159, Re = 5.05; Film 11. 4–1,Ta = 254, Re = 6.4; Film 12. 3V, Ta = 207, Re = 10.5.

A three-segment electrodiffusion (ED) probe was used to measure wall shear rate vector magnitude and its axial and azimuthal components at the wall of the outer cylinder. The vortices passed along the probe flush mounted in the wall and created their imprint in a form of segment currents. The probe is on the upper left side of the cylinder at a distance of 205 mm from the bottom, see S1 Film and Fig 4.

### Flow regimes and wall shear rate variation

**Fig 5:**
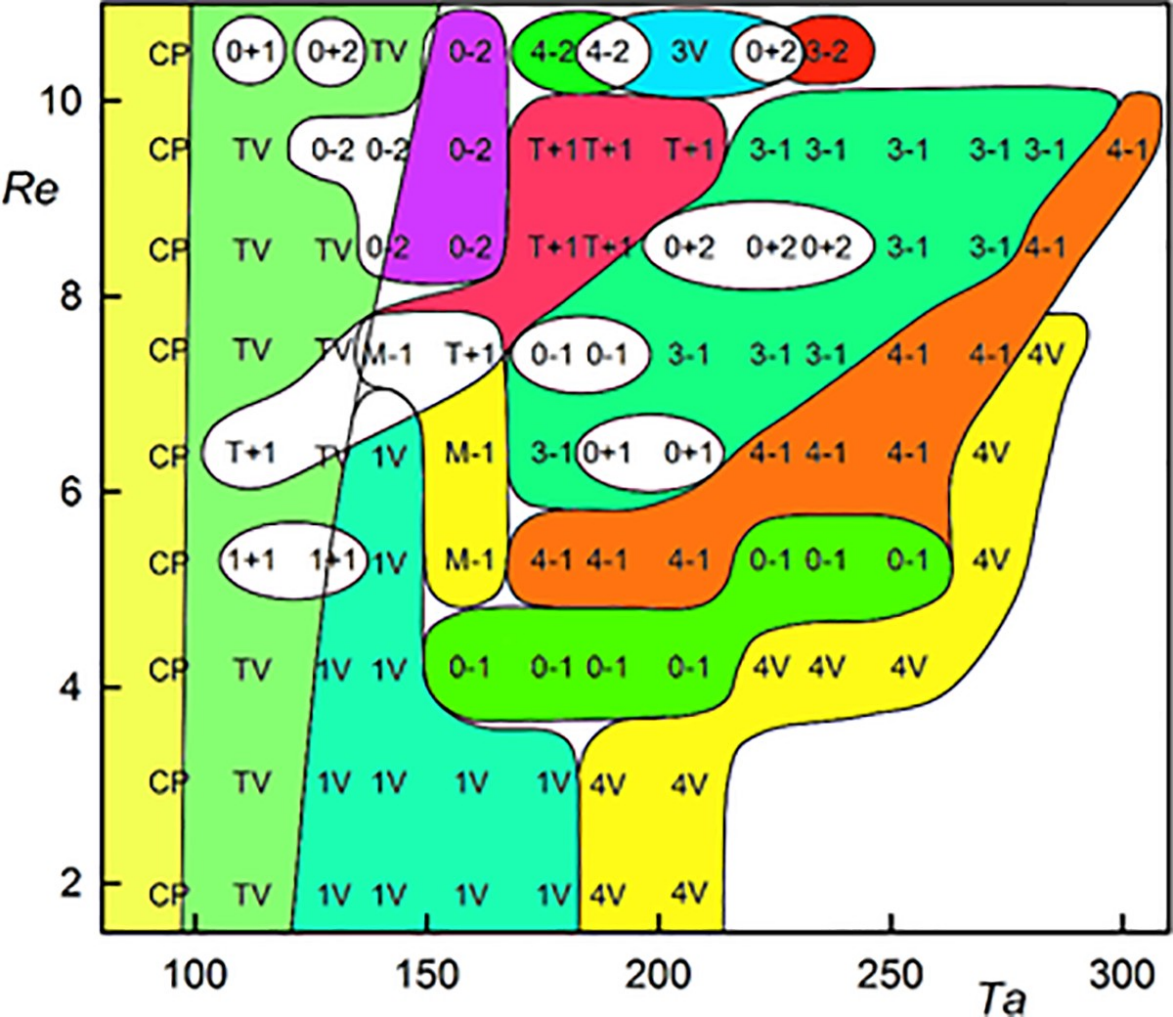
Regimes observed at different Ta and Re numbers. Acronyms represent measurement points. The curves sketched between flow regimes are a visual guide, but should not be constructed as sharp boundaries. 0–1 –standing simple helix winding in the same direction as the base flow. 0–2 –standing double helix winding in the same direction as the base flow. 0+1 –simple helix winding in the opposite direction to the base flow. 0+2 –double helix winding in the opposite direction to the base flow. 1+1 –simple helix winding in the opposite direction to the base flow with one azimuthal wave. 3–1 –standing simple helix winding in the same direction as the base flow with three azimuthal waves. 4–1 –standing simple helix winding in the same direction as the base flow with four azimuthal waves. 3–2 –standing double helix winding in the same direction as the base flow with three azimuthal waves. 4–2 –standing double helix winding in the same direction as the base flow with four azimuthal waves. 1V –vortex flow with one predominant azimuthal wave with sudden variation of slope. 3V –vortex flow with three azimuthal waves. 4V –vortex flow with four azimuthal waves. CP–Couette Poiseuille flow. M-1 –simple helix winding and moving in the same direction as the base flow. T+1 –simple helix winding in the opposite direction to the base flow interpenetrated with Taylor vortices. TV–Taylor vortices moving with the axial flow.

Different flow regimes found in the investigated range of *Re* ϵ (1.9, 10.5) and *Ta* ϵ (94, 319) are presented in Fig 5. The downhole drilling application has similar range of operating regime as those studied in the present investigation [40]. Each symbol in Fig 5 stands for parameters of a single experiment. The experiments were carried out at least in triplicate. The white areas denote the regions where several regimes were found. All regimes were established using the filling procedure, which consists in filling the annular gap under rotation of the inner cylinder. The flow structures developed in the presence of a free surface and rose with the mean axial flow. The filling experiments resulted in the flow structures which were also observed in the start experiment [14].

**Fig 6:**
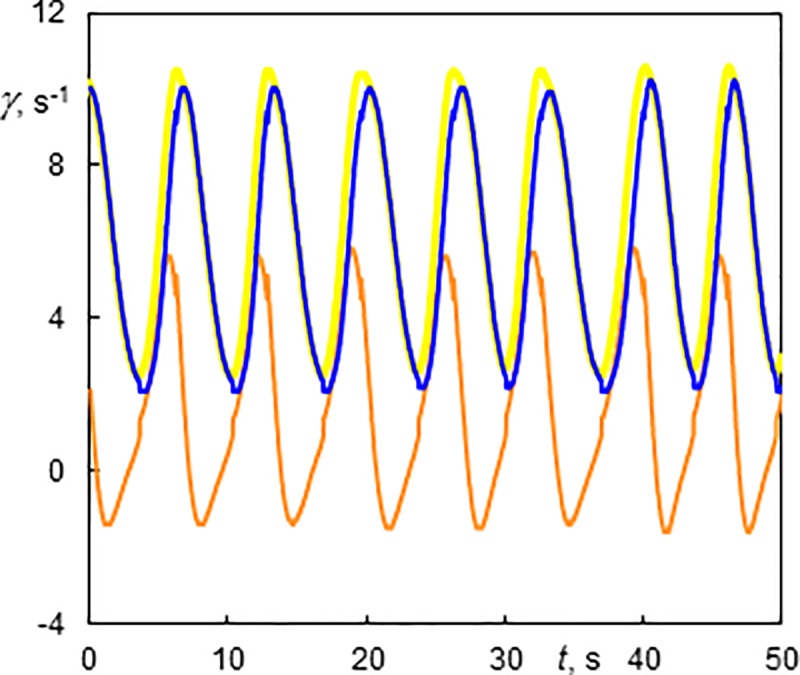
History of wall shear rate components at the outer cylinder of a Taylor vortex flow (TV) at Ta = 142 and Re = 10.5. The light thick line (yellow online)–total wall shear rate; the darkest line (blue online)–the azimuthal component; and the shadowy line (orange online)–the axial component. The 10 s time corresponds to 18.9 mm of the axial scale.

Laminar Couette-Poiseuille flow (CP) existed at *Ta* = 94 over the whole interval of investigated *Re*. Esser and Grossmann [41] calculated a critical number of *Ta*_*c*_ = 97.6 for the stability of the Couette flow (*Re* = 0) and Recktenwald et al. [16] gave *Ta*_c_ = 95.5 and 95.9 for *Re* = 0 and 10, respectively. Taylor vortex flow (TV) means the standard toroidal Taylor vortices translated at an axial velocity that is slightly higher than the mean velocity of the base flow. This flow mainly occurred at low *Ta*. An example is shown in S1 Film (*Ta* = 142, *Re* = 10.5), see Fig 4A for the snapshots of the films. Disorder flow was present at the bottom and regular Taylor vortices started at a distance of 80 mm from the bottom. The vortices had an axial wavelength of 13.2 mm (*s/d* = 1.09) and they moved in the direction of axial flow at a speed of 1.89 mm.s^-1^. The ratio of the axial phase speed to the axial mean base flow velocity *u*/*u*_m_ was 1.09. The corresponding wall shear rate of TV flow is shown in Fig 6. Using the axial velocity of vortex, the time scale can be transformed into an axial scale. Each period (6.71 s in Fig 6) corresponds to the passage of one vortex pair. The shadowy line (orange online) denotes to the axial component, which has a non-zero mean value. The azimuthal component (the darkest line—blue online) had values between 2 and 10 s^-1^. This component was always positive as it reflected rotation (of structures) about cylinder axis. The total wall shear rate (see the light thick line—yellow online in Fig 6) did not differ much from the azimuthal component because the values of the axial component were low with respect to the azimuthal component. This is the reason the total wall shear rate was not depicted in the followinγ figures. The maximum and minimum values of the azimuthal component of wall shear rate lay in the outflow and inflow regions of Taylor vortices, respectively. In the both regions, the axial component had a value related to the phase speed of vortices, i.e. about 2 s^-1^. The phase speed of Taylor vortices can be obtained either from ED currents or from film. The axial wavelength of Taylor vortices (*Ta* = 142, *Re* = 10.5), 13.2 mm, was obtained from the correlation of the currents passing through small electrodes shifted by 6 mm in vertical direction (Fig 3). The frequency of vortex passing over the probe, 6.71 s^-1^, was obtained from the current autocorrelation or Fig 6. The velocity 1.97 mm.s^-1^, calculated from these two data, correspond well with the phase speed of 1.9 mm.s^-1^ determined from S1 Film.

Vortex flow with one predominant azimuthal wave with a sudden variation of slope (1V) was present at low *Ta* and *Re* (*Ta* ≤ 189 and *Re* ≤ 6.4) (S2 Film, *Re* = 4.2 and *Ta* = 142). There can be seen also three small waves between two predominant waves. It is not clear from the film if the flow had the form of closed toroidal vortices (rings) or a helix. However, the visually observed axial phase speed of the structures was about 1.13 times the mean velocity of the axial flow, which corresponds to the vortices. The vortices had a wave period of 18 s and an axial wavelength and velocity of 9.7 mm (*s/d* = 0.8) and 0.77 mm.s^-1^, respectively.

**Fig 7:**
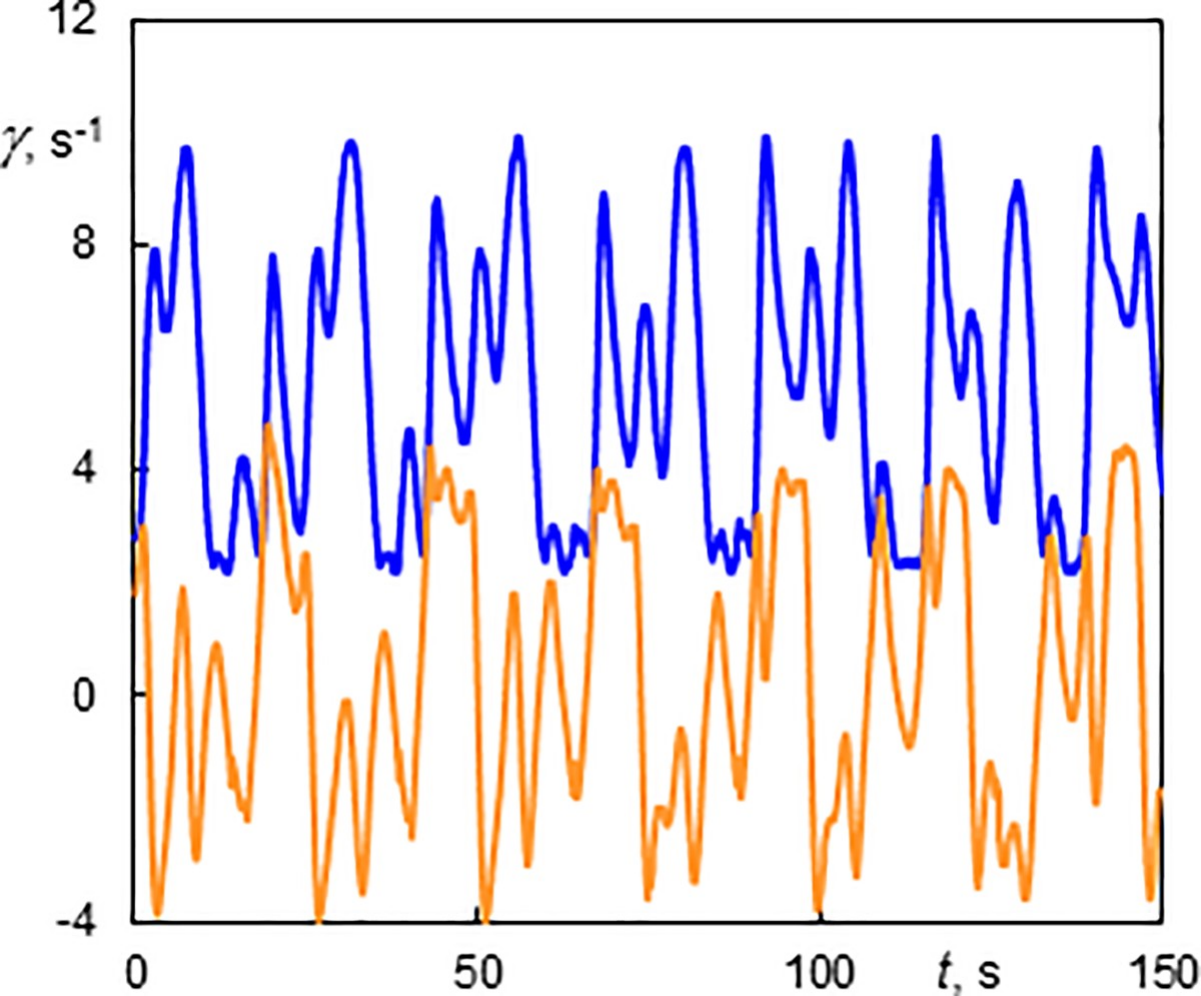
History of wall shear rate components at the outer cylinder in a vortex flow with one azimuthal wave having a sudden variation of slope (1V) at Ta = 142 and Re = 3. The light line (orange online)–the axial component; the dark line (blue online)–the azimuthal component. The 10 s time corresponds to 5.26 mm of the axial scale.

The components of wall shear rate in the regime with one predominant wave with sudden variation of slope (1V) are shown in Fig 7 (*Re* = 3 and *Ta* = 142). The axial wavelength of vortices was 12.1 mm (*s/d* = 1). The patterns of the wall shear rate components were chaotic. Nevertheless, we can discern a long period of 25.2 s which corresponds to the passage of a Taylor vortex and a short period of 6.3 s corresponding to waves. The passage of small waves between two consecutive big waves was presented in S2 Film. A vortex flow with one azimuthal wave was also described by Altmeyer et al. [22].

**Fig 8:**
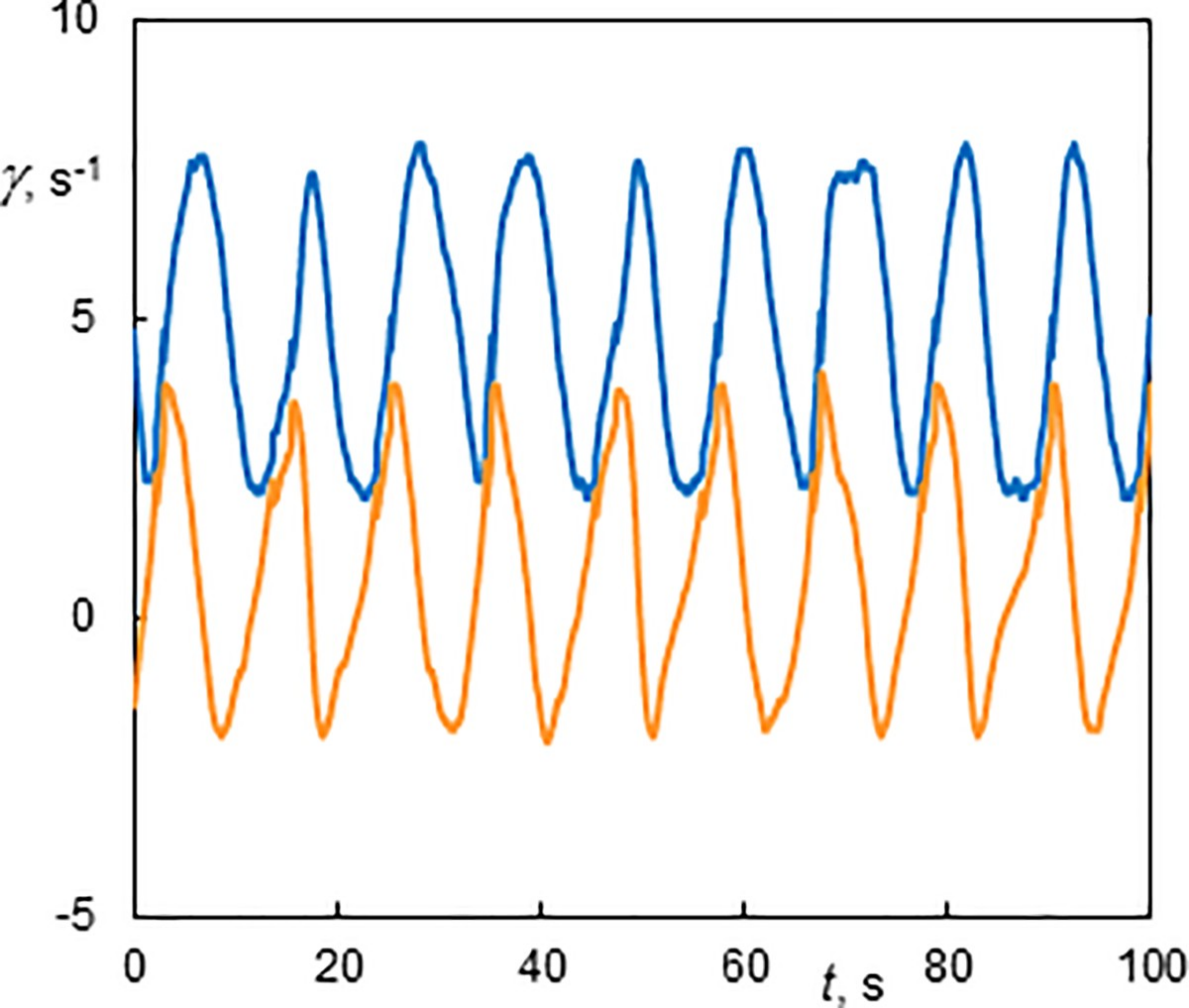
History of wall shear rate components at the outer cylinder of TV flow at Ta = 142 and Re = 5.3. The light line (orange online)–the axial component; the dark line (blue online)–the azimuthal component. The 10 s time corresponds to 9.32 mm of the axial scale.

With an increase in the Reynolds number, the small waves disappeared and the course of the wall shear rate became more regular. The TV flow is shown in Fig 8 (*Re* = 5.3, *Ta* = 142), but it can alternate with 1V or 1+1 flow (see Fig 5). The maxima of the azimuthal component were lower than at *Re* = 3 due to the stabilizing effect of the axial flow. The axial wavelength of vortices was 9.4 mm (s/*d* = 0.78).

Starting from middle values of *Re* and low *Ta*, a helix winding in the opposite direction to the base flow was observed, with one wave on the perimeter characterized by a large variation of the slope (1+1) and a period of 18 s (S3 Film, *Ta* = 130, *Re* = 5.3). The visually observed axial velocity of the helix was 1.83 times the mean axial base flow velocity, which corresponds to a helix winding in the opposite direction to the base flow. The helix had an axial wavelength of 11.6 mm (*s/d* = 0.96).

At *Re* = 10.5, the waves disappeared and the flow was in perfect helices, winding in the opposite direction to the base flow. The axial flow stabilized these helices. There were single helices (0+1) at *Ta* = 112 (S4 Film and double helices (0+2) at *Ta* = 130 (S5 Film. The axial wavelength was 10.9 mm (*s/d* = 0.9) and 12.6 mm (*s/d* = 1.04) for the 0+1 and 0+2 helices, respectively. The 0+1 and 0+2 helices moved upward at an axial phase speed of 2.61 and 3.51 mm.s^-1^, which was one and half and twice the velocity of the mean axial base flow, respectively. When the axial flow was stopped at 18 s (S6 Film, see playtime of the Windows media player or another player), the helix still moved upwards but at a lower axial velocity of 0.56 mm.s^-1^ (*u*/*u*_m_ = 0.32); this was due solely to the rotation effect. Hence the rotation effect can be decoupled from the axial flow.

**Fig 9:**
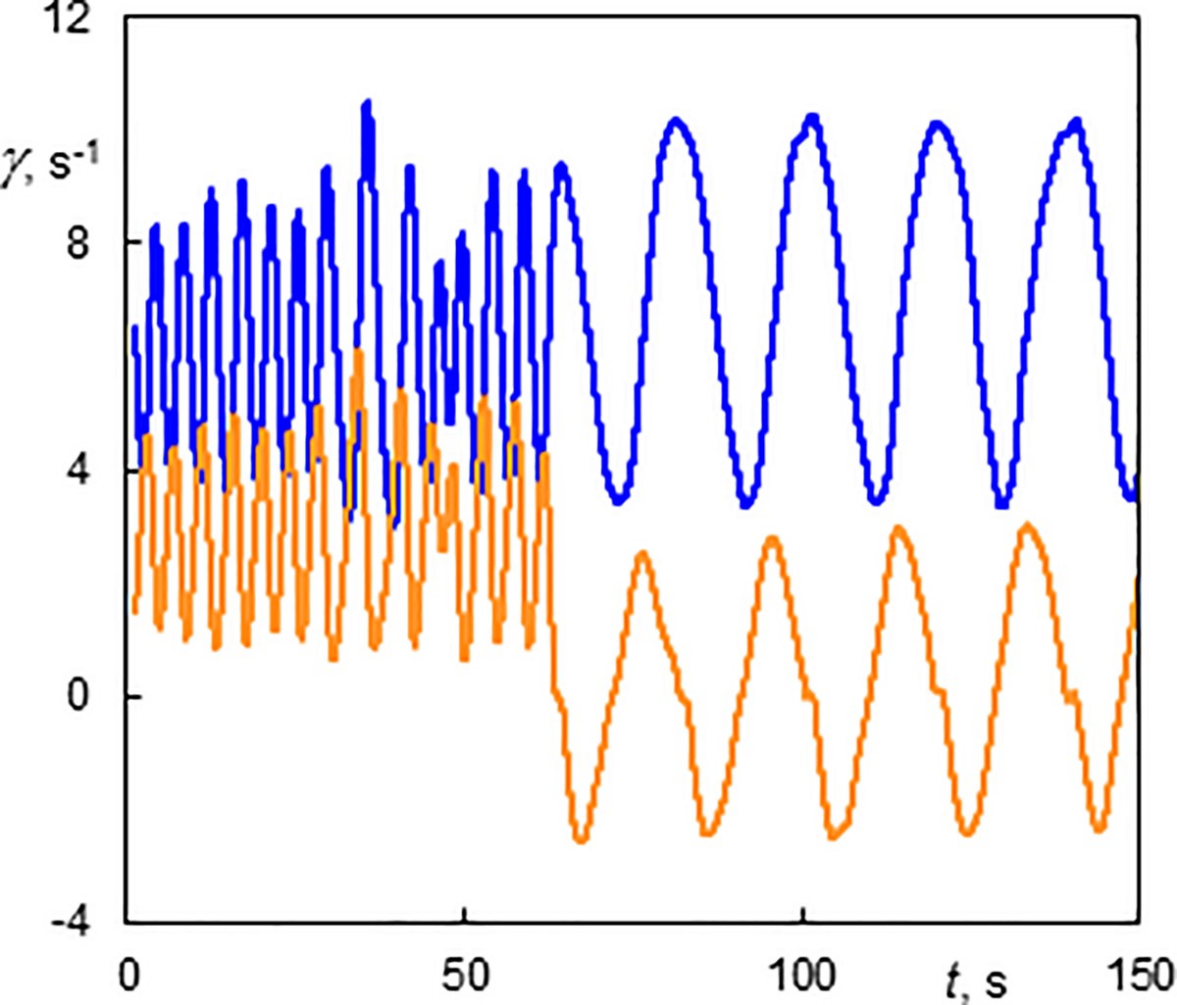
Wall shear rate components at the outer cylinder of a helix winding in the opposite direction to the base flow (Ta = 112, Re = 10.5, 0+1). The axial flow was stopped at 60 s.The light line (orange online)–the axial component; the dark line (blue online)–the azimuthal component.

The wall shear rate of a fully developed single helix winding in the opposite direction to the base flow (*Re* = 10.5, *Ta* = 112, 0+1) is depicted in Fig 9. The first part of the figure was recorded with axial flow. The period was short due to the rapid passage of the helix along the probe. The mean value of the axial component reflects the axial phase speed of the helix. In the second part, the axial base flow was stopped but the helix continued its rotation. The long period matches the helix rotation without axial motion. This regime was shown in S4 Film and the effect of ceasing axial flow in S6 Film. It should be emphasized that the helical structure persists in its original form at least 100 s after turning off the axial flow but keeping the rotation of the inner cylinder.

The helices winding in the opposite direction to the base flow also occurred at higher *Ta* but they were interpenetrated with a wavy vortex flow (T+1) (S7 Film and Fig 4B, *Ta* = 159, *Re* = 7.4). At 7 s and a distance of 120 mm the helix was penetrated by a wavy vortex flow (S7 Film. The same phenomenon was observed at 28 s and 140 mm.

Double helices winding in the same direction as the base flow sometimes occurred at higher *Ta* (S8 Film and Fig 4B, *Ta* = 142, *Re* = 8.5). The helices appeared as fixed spiral tubes through which the liquid passed. Due to the rotation effect the phase speed of helices was zero (0–2). The axial speed of the helices, which results from the base axial flow, was compensated by the downward velocity associated with the rotation effect. The stagnation of helices winding in the same direction as the base flow, i.e. the reason the axial velocity caused by the rotation effect is equal in absolute value to the velocity due to axial base flow, has never been explained. These helices were formed at the bottom while the annular gap was being filled with the electrolyte. At given conditions, the helices were always stable at least until half way up the cylinder. Occasionally they filled the whole cylinder to the top. When the axial flow was stopped (S6 Film at 15 s), the helices moved downward at a phase speed of -1.53 mm.s^-1^ (*u*/*u*_m_ = -1.09). The helices winding in the same direction as the base flow never occurred in the start experiment. The filling experiment with the 0–1 helices is shown in S9 Film (*Ta* = 159 and *Re* = 5.05). In order to show the filling process over the whole length of the cylinder in a reasonable time, several parts of the movie were cut. The jumps of the free surface correspond to the edited sections. The ascending velocities of the free surface 0.82 mm.s^-1^ corresponded to the mean axial flow. An annular vortex is observed below the free surface and the helix ends below this vortex.

**Fig 10:**
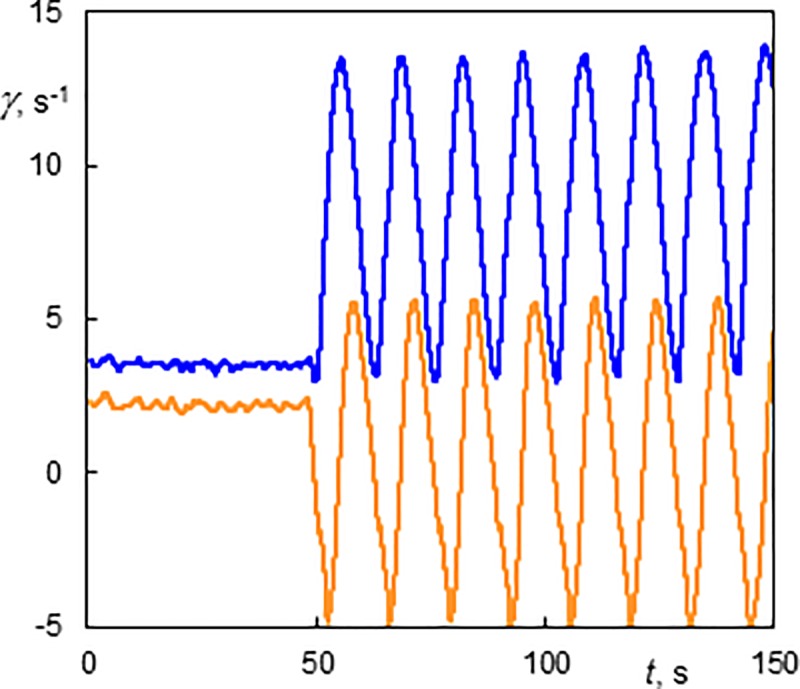
History of wall shear rate components at the outer cylinder of the standing helix winding in the same direction as the base flow (Ta = 177, Re = 4.2, 0–1). The axial flow was stopped at 50 s. The light line (orange online)–the axial component; the dark line (blue online)–the azimuthal component.

The wall shear rate of a standing helix winding in the same direction as the base flow (*Ta* = 177, *Re* = 4.2, 0–1) is shown in Fig 10. The first part presents a standing helix exhibiting an almost constant wall shear rate. The values of wall shear rate depend on the helix position with respect to the electrodiffusion probe. When the axial flow was stopped, only the helix rotation persisted. The axial wavelength was 10.3 mm (*s/d* = 0.85). The periodic wall shear rate corresponds to its distribution along the perimeter. The values of the shear rate components of the standing helix are in the domain of those measured without axial flow. This type of flow was shown in S9 Film. Lueptow et *al*. [11] observed stationary helical vortices in a small domain around *Ta* = 150 and *Re* = 5. As there was a little difference in the gap width (η = 0.83 in [11]), this result matches well our observation.

**Fig 11:**
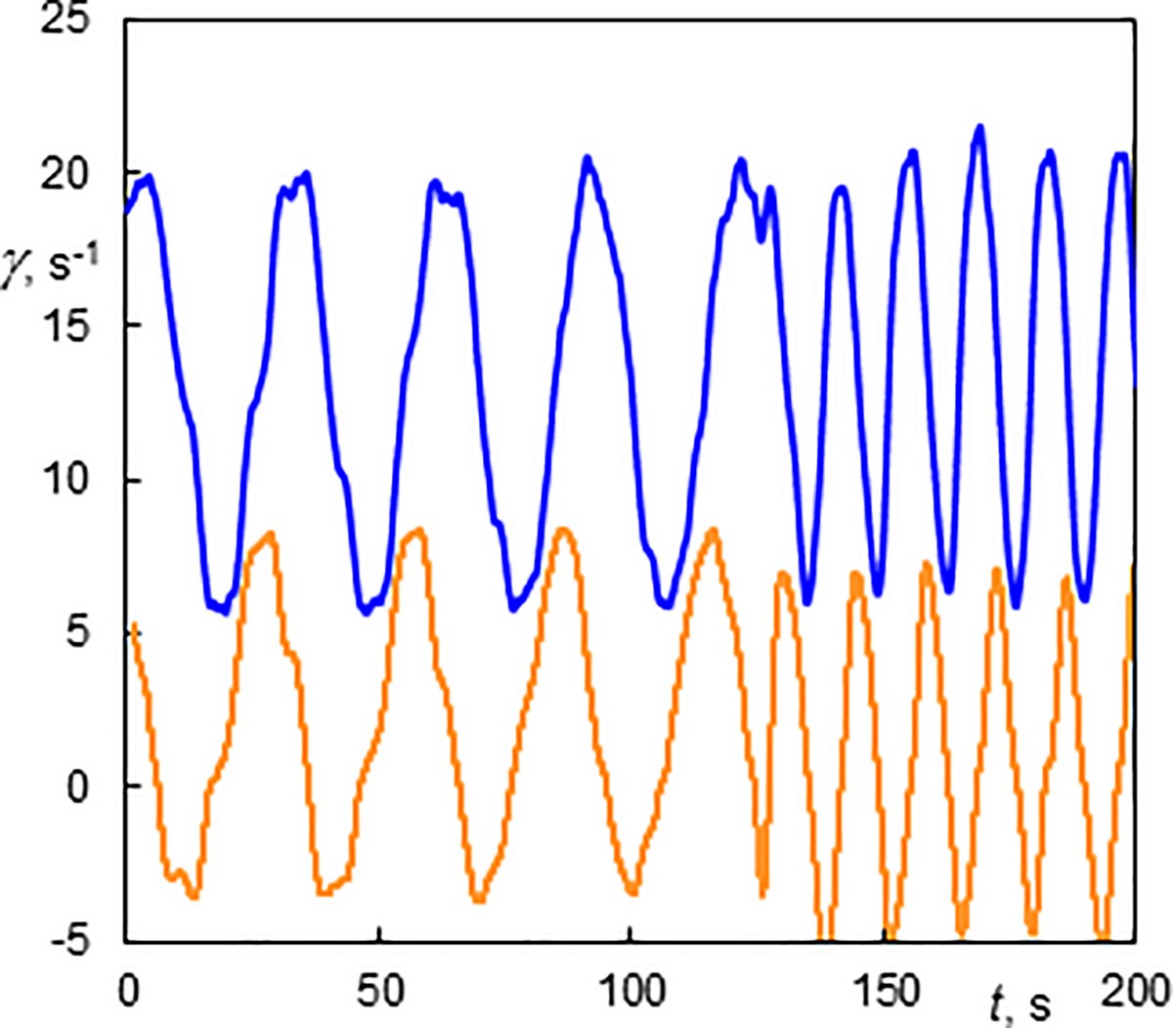
History of wall shear rate components at the outer cylinder of a helix winding in the same direction as the base flow (Ta = 159, Re = 5.05, M-1). The axial flow was stopped at 125 s. The light line (orange online)–the axial component; the dark line (blue online)–the azimuthal component.

The helices winding in the same direction as the base flow sometimes moved upward (M-1) (S10 Film, *Ta* = 159 and *Re* = 5.05). This helix had an axial wave length of 8.82 mm (*s/d* = 0.73) and a velocity 0.3 mm.s^-1^ (*u*/*u*_m_ = 0.36). When the axial flow was stopped at 37 s the helix moved downwards at a velocity of 0.61 mm.s^-1^ (*u*/*u*_m_ = -0.73). The corresponding wall shear rate components of a helix rising at a slow velocity had a long period (29.4 s) while the rotating helix without axial motion resulted in a short period (13.9 s), see Fig 11. The mean axial component of *γ* is finite when the axial base flow is installed, otherwise is zero.

**Fig 12:**
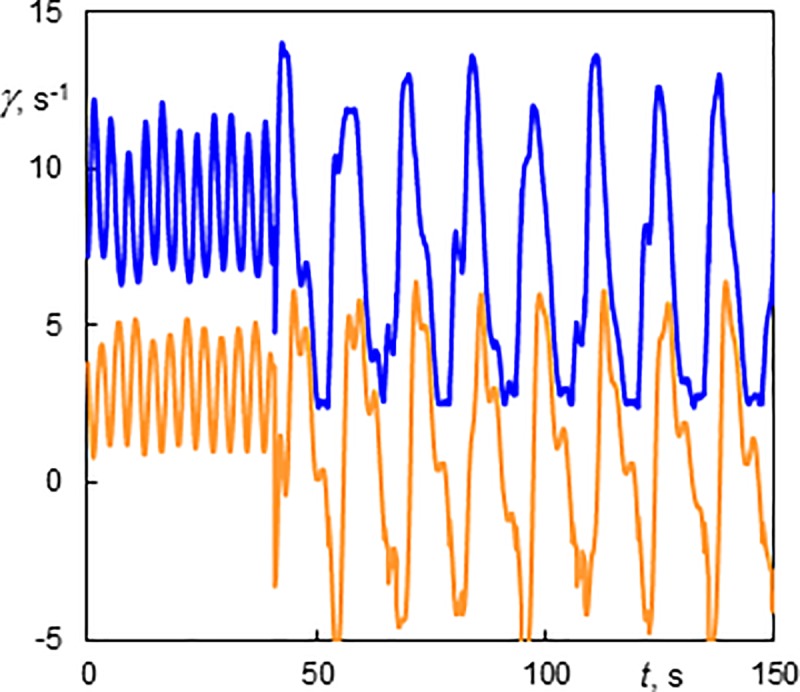
History of wall shear rate components at the outer cylinder in a standing wavy helix winding in the same direction as the base flow (Ta = 177, Re = 5.1, 4–1). The axial flow was stopped at 40 s. The light line (orange online)–the axial component; the dark line (blue online)–the azimuthal component.

The helices became wavy with four azimuthal waves (4–1) at higher *Ta* numbers (S11 Film, *Ta* = 254, *Re* = 6.4). This steady helix had an axial wavelength of 12.6 mm (*s/d* = 1.04). The waves were not in phase along the axis. When the axial flow was stopped (at 10 s in S11 Film),the wavy structure was conserved and the helix moved downward at a velocity of 1.08 mm.s^-1^ (*u*/*u*_m_ = -1.04). The wall shear rate components of the standing wavy helix winding in the same direction as the base flow (4–1, *Ta* = 177, *Re* = 5.1) are shown in Fig 12. In the first part, the azimuthal waves were superposed on constant values of the wall shear rate components. The mean value and amplitude of oscillations of the azimuthal component depend on the position of the helix with respect to the probe. The high frequency in the first part is due to the passage of four azimuthal waves per one helix revolution along the probe. When the axial flow was stopped (at 40 s in Fig 12), the signal period of about 10 s corresponds to the helix rotation divided by the number of helix (a single helix in this case). The azimuthal waves can be discerned as perturbations on the signals. The ratio of the periods with and without axial flow is about 4, which corresponds to 4 azimuthal waves and a single helix. The axial wavelength is 12.7 mm (*s/d* = 1.05). The regime 4–1 was observed in a wide range of experimental conditions, see Fig 5.

**Fig 13:**
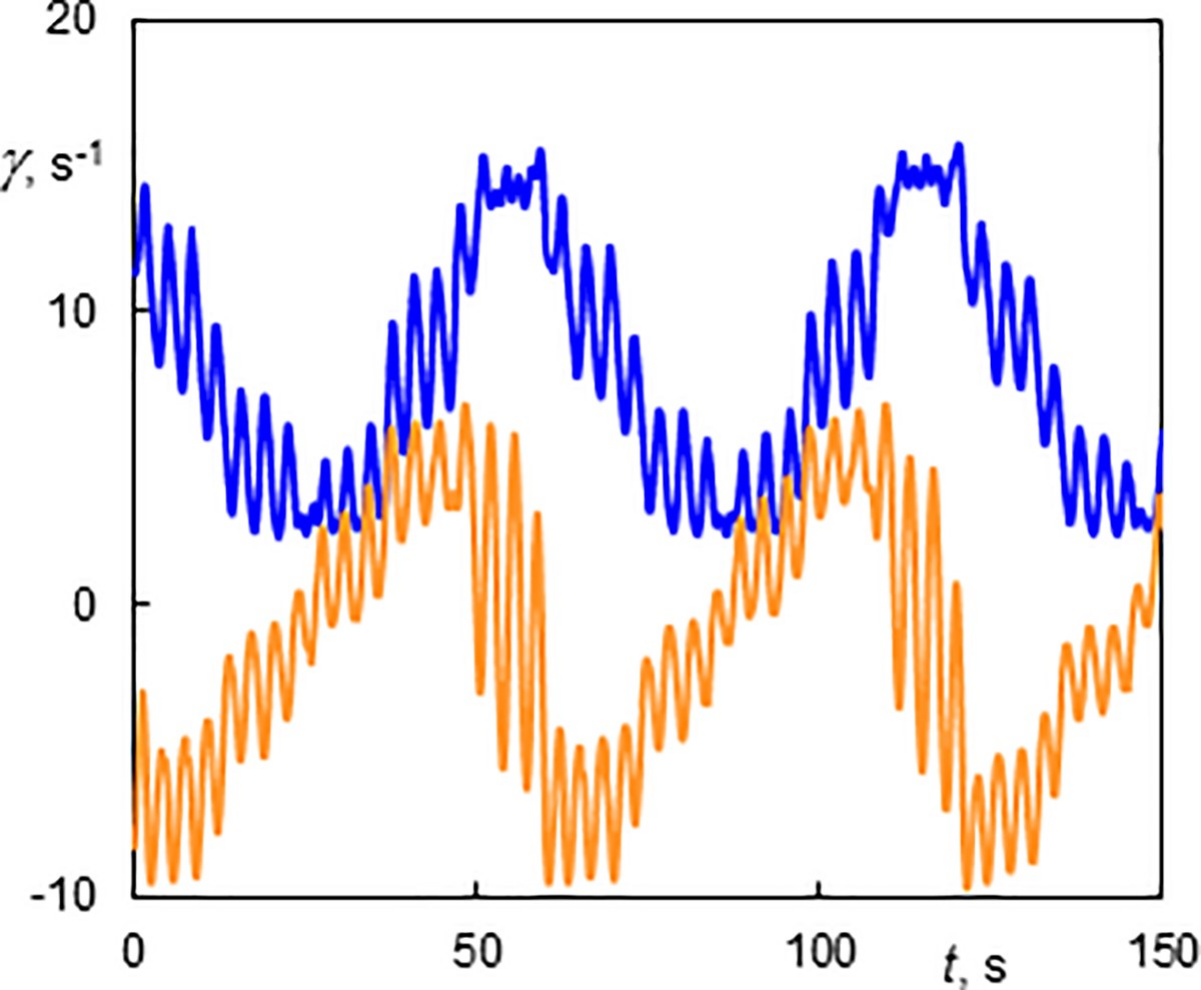
History of wall shear rate components at the outer cylinder in a wavy vortex flow (Ta = 207, Re = 1.9, 4V). The light line (orange online)–the axial component; the dark line (blue online)–the azimuthal component. The 10 s time corresponds to the 20.4 mm vortex axial scale.

Wavy toroidal vortices were observed at higher *Ta* numbers and at *Re* lower than 8.5. The only exception were the toroidal vortices with 3 azimuthal waves (3V) observed at *Re* = 10.5 and *Ta* = 207 (S12 Film. The vortices had an axial wavelength of 9.1mm (*s/d* = 0.75) and a velocity of 1.93 mm.s^-1^ (*u*/*u*_m_ = 1.11). The amplitude of the azimuthal waves was not uniform, and increased as a function of axial distance from the bottom. Azimuthal waves on Taylor vortices are clearly visible at low Reynolds numbers (Fig 13, *Re* = 1.9, *Ta* = 207, 4V). They can be seen on both wall shear rate components. The azimuthal component of wall shear rate had the least fluctuations at the inflow and outflow. The fluctuations of the axial component were maximal at the outflow. Lueptow et al. [11] observed wavy toroidal vortices at *Ta* >125 and *Re* < 5.

Different regimes were observed at the same *Ta* and *Re* numbers. White color in Fig 5 corresponds to the areas where several regimes can be present. A moving helix (M-1) and a helix interpenetrated with Taylor vortices (T+1) were seen at *Ta* = 142 and *Re* = 7.4. Double helix winding in the opposite direction to the base flow (0+2) sometimes developed at *Ta* = 224 and *Re* = 10.5 and at other times a standing wavy double helix (3–2) was observed under these conditions. Simple helices winding in the opposite direction to the base flow (0+1) and wavy helices winding in the same direction as the base flow (3–1) were observed alternatively at *Ta* = 189 and *Re* = 6.4. There are very subtle unknown flow conditions which switch regimes. Using numerical simulations, Altmeyer et al. [17] found an island of double helices winding in the same direction as the base flow in the region of double helices winding in the opposite direction to the base flow for *Ta* of about 175 and *Re* = 6.

### Wall shear stress and frequency analysis

**Fig 14:**
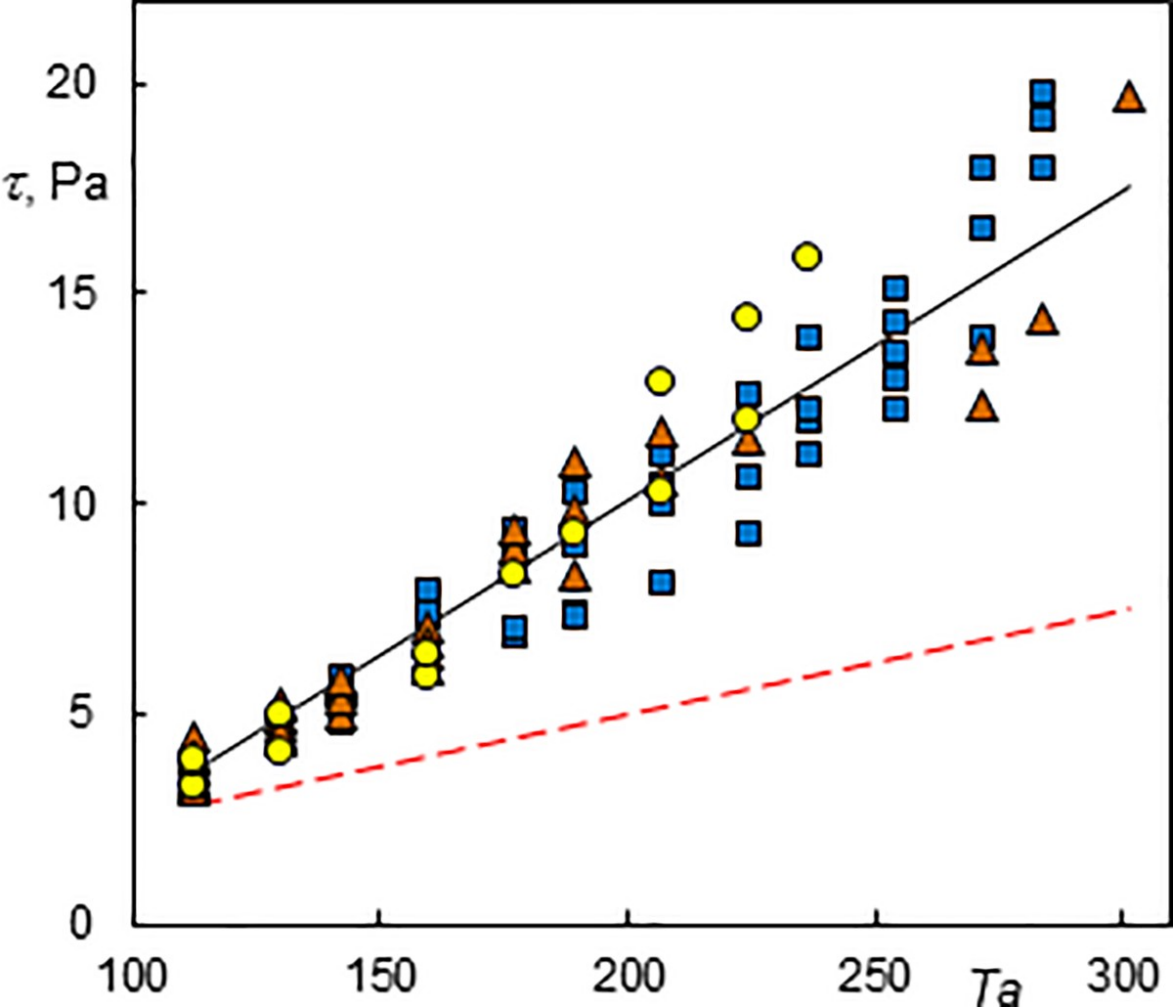
Azimuthal component of the wall shear stress. The dashed light line (red online) represents a supercritical Couette flow and the black line a numerical simulation of a supercritical Taylor vortex flow [35]. Triangles represent Taylor vortices, rectangles indicate helices winding in the same direction as the base flow and circles correspond to helices winding in the opposite direction to the base flow.

**Fig 15:**
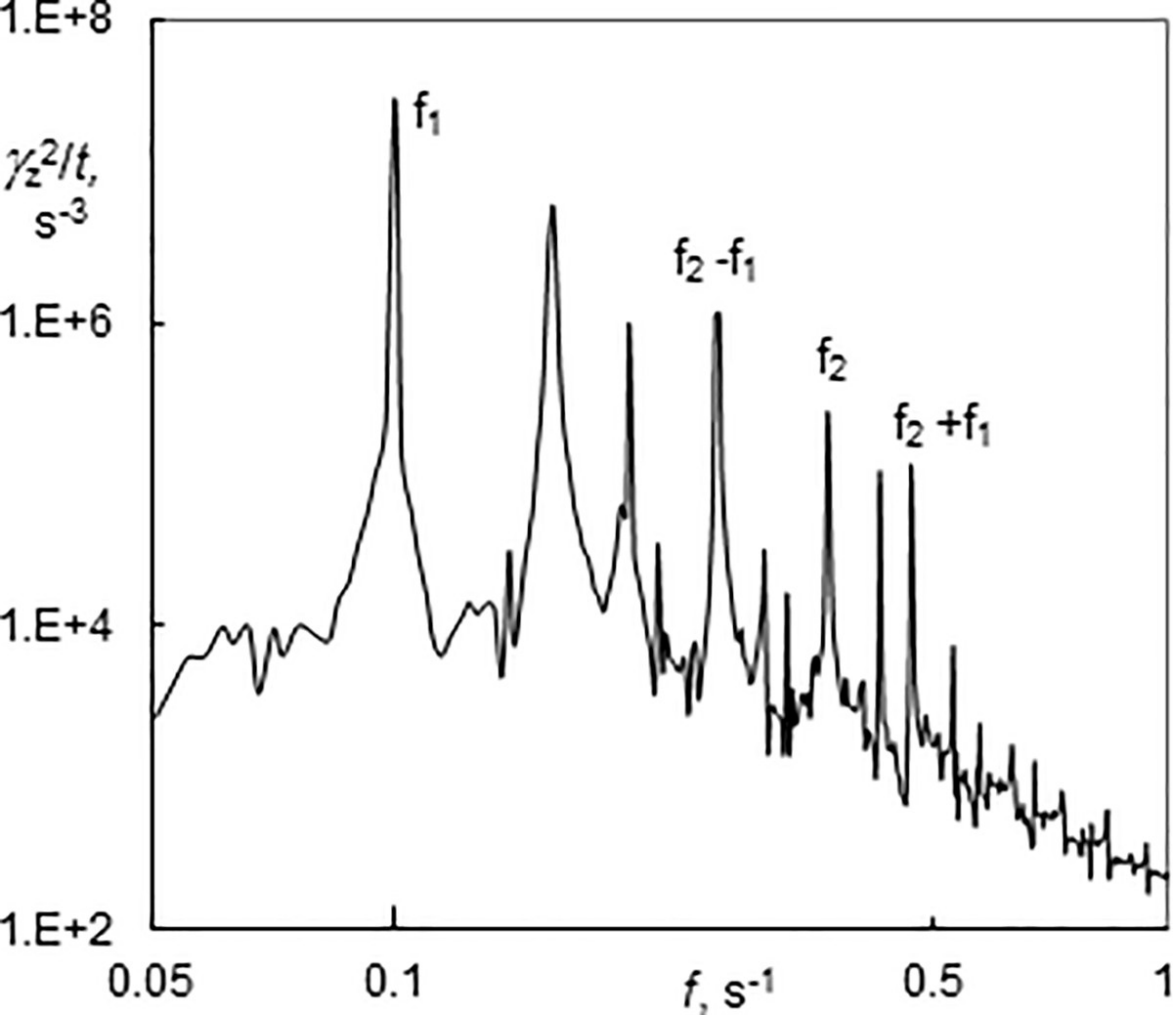
Power spectrum of wavy helices winding in the same direction as the base flow (3–1) after stopping the axial flow (Ta = 254, Re = 8.5). f_1_ and f_2_ stand for frequency of helix rotation and passage of azimuthal waves, respectively.

Using the dynamic viscosity of the solution (*μ* = 1.025∙10^−3^ Pa s), the wall shear stress was calculated from the mean value of the azimuthal component of wall shear rate. Wall shear stress has been found to be a function of *Ta* but no significant dependence on *Re* was observed (Fig 14). The results are scattered around the line which corresponds to the numerical simulations without axial base flow using Fluent under the assumption that Taylor vortices are present at supercritical Taylor numbers [35]. The wall shear stress at low *Re* fits well with this line, while most values for moderate *Re* (5.1–7.4) were below it and the values for higher *Re* (8.5–10.5) were above it. The scatter of τ is due to different flow regimes. For example, two measurements carried out during a 20 minute interval at *Re* = 8.5 and *Ta* = 254 and 272 (rectangles in Fig 14) were both present in the regime of a standing wavy simple helix but the difference in *τ* (17.6–14.3 = 3.3 Pa) was higher than the difference corresponding to the variation of *Ta*. It was impossible to distinguish any difference visually. However, the number of azimuthal waves on the helix at *Ta* = 254 was equal to three while the helix at *Ta* = 272 had four waves. It can be concluded that the mean wall shear stress increased with the number of azimuthal waves. There are also differences in the power spectrum of the azimuthal components of the wall shear rate. The power spectrum at *Ta* = 254 had several peaks (Fig 15). The frequencies f_1_ and f_2_ correspond to the rotation of a helix and the passage of azimuthal waves, respectively.

**Fig 16:**
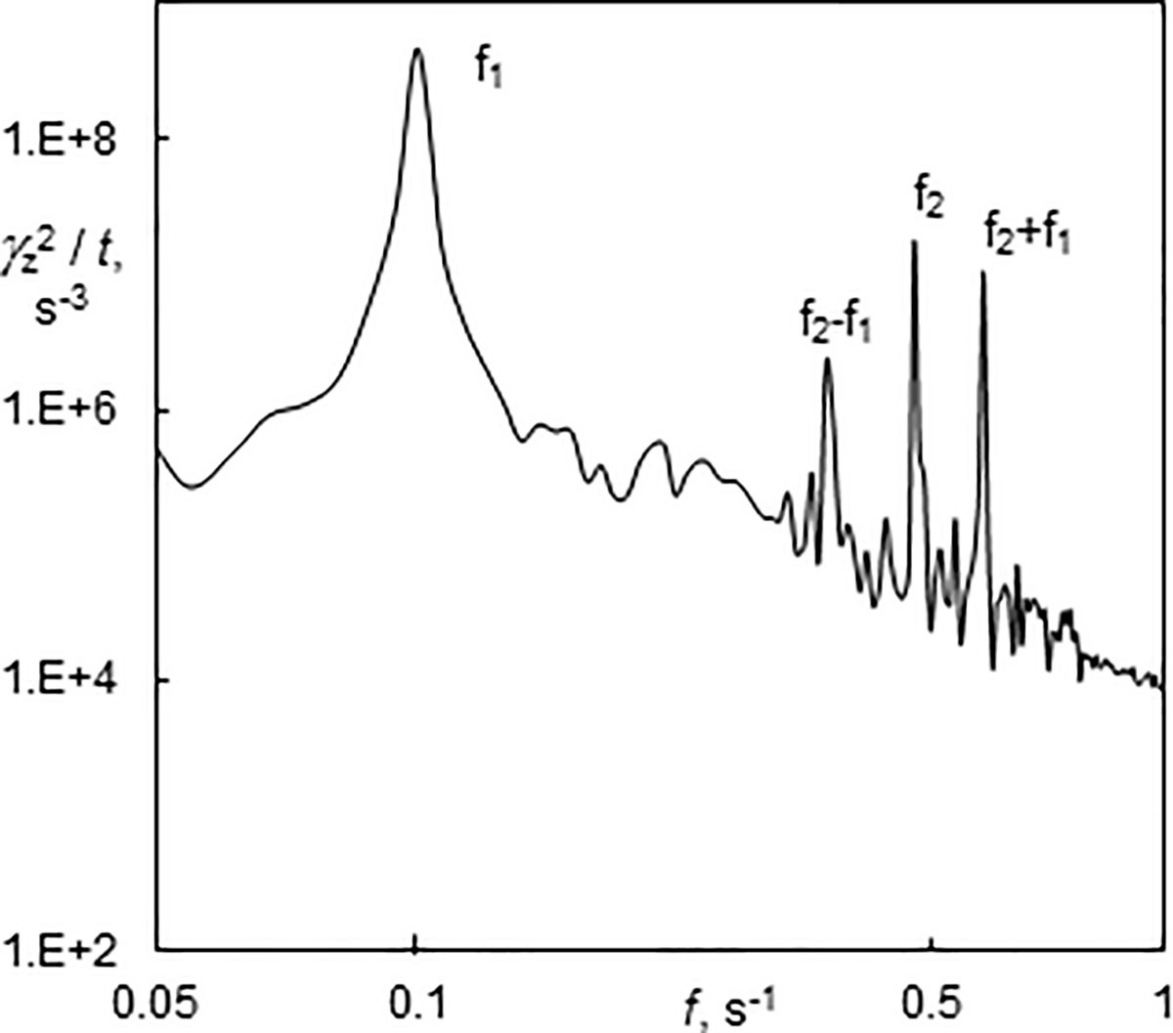
Power spectrum of wavy helices winding in the same direction as the base flow (4–1) after stopping axial flow (Ta = 272, Re = 8.5). f_1_ and f_2_ represent the frequency of helix rotation and the passage of azimuthal waves, respectively.

The power spectrum at *Ta* = 272 displayed only peaks, corresponding to f_1_ and f_2_ (Fig 16). The amplitude of the axial wall shear rate component at *Ta* = 272 was greater than at *Ta* = 254, which means that the energy dissipation was more intensive. ED probes can give us more detailed information about flow patterns than torque measurements. It should be emphasised that ED probes measure local values. The flow patterns are not often uniform along the length of a cylinder. This fact can also contribute to the differences in *τ*. Scattering can also be seen in the results published in the literature [13]. There is a lack of data on wall shear stress in Taylor-Couette-Poiseuille flow at low *Ta* numbers that could be used for comparison. Moreover, the standard torque measurements were carried out without distinguishing flow regimes [29]. The numerical simulations of Huang and Yang [13] showed that axial flow diminished wall shear stress.

## Conclusions

The flow dynamics of the large scale structures of a Taylor-Couette-Poiseuille flow were studied at low axial Reynolds and rotational Taylor numbers (*Re* ≤ 10.5, *Ta* ≤319). Simultaneous flow visualizations and polarographic measurements were conducted to investigate the flow patterns and their influence on both the axial and azimuthal components of the wall shear stress, with and without axial base flow. Complete map of flow regimes was presented.

The azimuthal component of the wall shear rate *γ* has the maximum at the outflow, where the value of the axial component corresponds to the mean axial velocity of the flow structure. The lowest value of the azimuthal component of *γ* was at the inflow, where the axial component had the same value as in the outflow. The azimuthal and axial components of wall shear rate measured by ED three-segment probes can elucidate the subtle differences in flow regimes which result in different values of wall shear stress. To the authors’ knowledge, these components of the wall shear rate*γ*of helices has never been measured in previous experimental investigations. It should also be noted that the ED probes provide a more detailed information about flow patterns than torque measurements found in the literature.

It was found that the axial phase speed of Taylor vortices and wavy toroidal vortices is about 1.12 times the mean base velocity. In the study of helical flow, the axial phase speed of structures is composed of the helix rotation effect and translation due to the base axial flow. The component of helix phase speed due to the base axial flow was approximately the same as that of the vortices, i.e. about 1.12 times the mean axial velocity. The total phase speed of helices winding in the opposite direction to the base flow was within the interval 1.5–2. The helices winding in the same direction as the base spiral flow were stationary. The reason why the phase speed of these helices is zero has never been explained and this phenomenon has never been emphasized in the literature.

## Supporting information

S1 Film Taylor vortices moving with the axial flow. Ta = 142, Re = 10.5.
(MOV)

S2 Film Vortex flow with one predominant azimuthal wave with sudden variation of slope. Ta = 142, Re = 4.2.
(MOV)

S3 Film Simple helix winding in the opposite direction to the base flow with one azimuthal wave. Ta = 130, Re = 5.3.
(MOV)

S4 Film Simple helix winding in the opposite direction to the base flow. Ta = 112, Re = 10.5.
(MOV)

S5 Film Double helix winding in the opposite direction to the base flow.Ta = 130, Re = 10.5. (MOV)

S6 Film Simple helix winding in the opposite direction to the base flow. Ta = 112, Re = 10.8.
(MOV)

S7 Film Simple helix winding in the opposite direction to the base flow interpenetrated with Taylor vortices. Ta = 159, Re = 7.4.
(MOV)

S8 Film Standing double helix winding in the same direction as the base flow. Ta = 142, Re = 8.5.
(MOV)

S9 Film Standing simple helix winding in the same direction as the base flow. Ta = 159, Re = 5.05.
(MOV)

S10 Film Simple helix winding and moving in the same direction as the base flow. Ta = 159, Re = 5.05.
(MOV)

S11 Film Standing simple helix winding in the same direction as the base flow with four azimuthal waves. Ta = 254, Re = 6.4.
(MOV)

S12 Film Vortex flow with three azimuthal waves. Ta = 207, Re = 10.5.
(MOV)

S1 File Nomenclature. (DOCX)
