# Supplementary material for: Experimental investigation of Taylor-Couette-Poiseuille flow at low Taylor and Reynolds numbers
Source: PLoS One. 2019 Apr 3;14(4):e0212728. doi: 10.1371/journal.pone.0212728 (PMC6447155; doi:10.1371/journal.pone.0212728)
Supplement: S1 File — (DOCX) [file pone.0212728.s013.docx]

**S1 File**

**Nomenclature**

*c*_0_ concentration of active ions in bulk

*D* coefficient of diffusivity

*d* gap between cylinders

*F* Faraday constant

*I* limiting diffusion current

*i* current density

*K*_j_ coefficient in Eq 5

*L* electrode length

*n* number of helix starts

*n*_j_ exponent in Eq 5

*R* electrode radius

*R*_1_ radius of inner cylinder

*R*_2_ radius of outer cylinder

*Re* Reynolds number, Eq 2

*k* number of electrons involved in electrochemical reaction

*s* cell spacing (axial wave length = 2 *s*)

*Ta*  Taylor number, Eq 1

*u* axial phase speed of flow structures

*u_m_* mean velocity of base axial flow

*x* distance measured from electrode front edge

*y* perpendicular distance from electrode

*φ* flow angle

*γ* wall shear rate

*ν* kinematic viscosity

Ω angular velocity of inner cylinder

*η* radius ratio

*τ* azimuthal wall shear stress

CP Couette-Poiseuille

ED electro-diffusion

TC Taylor-Couette

TCPF Taylor-Couette-Poiseuille flow

Start experiment consists in adjusting axial flow in a completely fulfilled annular gap and then slowly increasing the rotational rate of the inner cylinder.

Filling experiment consists in filling the annular gap under rotation of the inner cylinder.
